# Intermittent Hypoxia Mimicking Sleep Apnea Increases Passive Stiffness of Myocardial Extracellular Matrix. A Multiscale Study

**DOI:** 10.3389/fphys.2018.01143

**Published:** 2018-08-15

**Authors:** Núria Farré, Jorge Otero, Bryan Falcones, Marta Torres, Ignasi Jorba, David Gozal, Isaac Almendros, Ramon Farré, Daniel Navajas

**Affiliations:** ^1^Heart Failure Unit, Department of Cardiology, Hospital del Mar, Barcelona, Spain; ^2^Heart Diseases Biomedical Research Group, IMIM (Hospital del Mar Medical Research Institute), Barcelona, Spain; ^3^Department of Medicine, Universitat Autònoma de Barcelona, Bellaterra, Spain; ^4^Unitat de Biofísica i Bioenginyeria, Facultat de Medicina, Universitat de Barcelona, Barcelona, Spain; ^5^CIBER de Enfermedades Respiratorias, Madrid, Spain; ^6^Sleep Lab, Hospital Clinic of Barcelona, Barcelona, Spain; ^7^Institute for Bioengineering of Catalonia, The Barcelona Institute of Science and Technology, Barcelona, Spain; ^8^Department of Child Health, University of Missouri School of Medicine, Columbia, MO, United States; ^9^Institut d’Investigacions Biomèdiques August Pi i Sunyer, Barcelona, Spain

**Keywords:** atomic force microscopy, tensile test, heart mechanics, myocardial stiffness, ventricular strain, obstructive sleep apnea

## Abstract

**Background:** Tissue hypoxia-reoxygenation characterizes obstructive sleep apnea (OSA), a very prevalent respiratory disease associated with increased cardiovascular morbidity and mortality. Experimental studies indicate that intermittent hypoxia (IH) mimicking OSA induces oxidative stress and inflammation in heart tissue at the cell and molecular levels. However, it remains unclear whether IH modifies the passive stiffness of the cardiac tissue extracellular matrix (ECM).

**Aim:** To investigate multiscale changes of stiffness induced by chronic IH in the ECM of left ventricular (LV) myocardium in a murine model of OSA.

**Methods:** Two-month and 18-month old mice (*N* = 10 each) were subjected to IH (20% O_2_ 40 s–6% O_2_ 20 s) for 6 weeks (6 h/day). Corresponding control groups for each age were kept under normoxia. Fresh LV myocardial strips (∼7 mm × 1 mm × 1 mm) were prepared, and their ECM was obtained by decellularization. Myocardium ECM macroscale mechanics were measured by performing uniaxial stress–strain tensile tests. Strip macroscale stiffness was assessed as the stress value (σ) measured at 0.2 strain and Young’s modulus (*E*_M_) computed at 0.2 strain by fitting Fung’s constitutive model to the stress–strain relationship. ECM stiffness was characterized at the microscale as the Young’s modulus (*E*_m_) measured in decellularized tissue slices (∼12 μm tick) by atomic force microscopy.

**Results:** Intermittent hypoxia induced a ∼1.5-fold increase in σ (*p* < 0.001) and a ∼2.5-fold increase in *E*_M_ (*p* < 0.001) of young mice as compared with normoxic controls. In contrast, no significant differences emerged in *E*_m_ among IH-exposed and normoxic mice. Moreover, the mechanical effects of IH on myocardial ECM were similar in young and aged mice.

**Conclusion:** The marked IH-induced increases in macroscale stiffness of LV myocardium ECM suggests that the ECM plays a role in the cardiac dysfunction induced by OSA. Furthermore, absence of any significant effects of IH on the microscale ECM stiffness suggests that the significant increases in macroscale stiffening are primarily mediated by 3D structural ECM remodeling.

## Introduction

Obstructive sleep apnea (OSA) is a highly prevalent respiratory disease, affecting patients across the whole human life span from infants to the elderly (Peppard et al., 2013; Heinzer et al., 2015). OSA is characterized by repetitive obstructions in the upper airway causing periodic apneas, with consequent increases in negative intrathoracic pressure swings that result in intermittent oxyhemoglobin desaturations in arterial blood which are usually terminated by arousals. All these challenges potentially induce mechanical stress, maladaptive transcriptional regulation, disruption of sympathetic outflow and alteration of multiple immunoregulatory pathways (Lévy et al., 2015). Owing to intermittent hypoxemia, all patient organs and tissues experience recurrent events of nocturnal hypoxia-reoxygenation (Almendros et al., 2010, 2011, 2013; Torres et al., 2014; Moreno-Indias et al., 2015), which in the case of severe OSA disease can occur >60 times per hour with nadir reductions in arterial oxygen saturation down to 60–70% (Ruehland et al., 2009; Lloberes et al., 2011). Given the multifactorial pathophysiological mechanisms leading to OSA, there is no specific treatment to prevent the abnormal collapsibility of the upper airway during sleep, and hence OSA is usually a chronic lifetime disease that is most frequently managed by applying continuous positive airway pressure (CPAP) via a nasal mask interface (Sullivan et al., 1981). The positive pressure applied by CPAP into the lumen of the upper airway splints its walls, and thus avoids upper airway collapse. This palliative mechanical treatment is very effective, but only when the patient is actually using the device. Unfortunately, adherence to CPAP is generally sub-optimal, with a non-negligible proportion of patients being intolerant or completely non-adherent to CPAP and the remainder using the device only for a portion of their sleep (Wozniak et al., 2014).

Chronic exposure to hypoxia-reoxygenation – either during the period preceding OSA diagnosis (usually several years) or in the context of poor or non-adherence to CPAP therapy after diagnosis – is a major perturbation that has been shown to significantly and independently increase both the morbidities and mortality of patients with OSA (Jordan et al., 2014), with these findings being further corroborated by a large number of mechanistic studies in experimental models of the disease (Davis and O’Donnell, 2013). Indeed, the specific pattern of high-frequency and high-amplitude tissue hypoxia-reoxygenation swings as seen in OSA patients triggers a cascade of inflammatory and oxidative stress pathways which then impose a large number of deleterious repercussions in organ systems, primarily affecting the cardiovascular, metabolic, and neurocognitive systems, while also adversely affecting cancer risk and prognosis (Gozal et al., 2016; Mokhlesi et al., 2016). This maladaptive response to chronic hypoxia/reoxygenation contrasts with the favorable adaptations observed when this hypoxia/reoxygenation stimulus is provided using divergent paradigms that promote pre-conditioning (Almendros et al., 2014; Mallet et al., 2018).

The cardiovascular system is particularly susceptible to OSA. Indeed, OSA has now been conclusively and independently associated with increased cardiovascular morbidity and mortality, with a spectrum of morbid phenotypes ranging from subclinical coronary atherosclerosis and systemic hypertension to ischemic coronary artery disease, stroke, peripheral artery disease, arrhythmias, and cardiac failure (Dong et al., 2013; Utriainen et al., 2014; Schaefer et al., 2015; Lee et al., 2016; Parati et al., 2016; Medeiros et al., 2017; Seo et al., 2017; Zhao et al., 2017). The mechanisms underlying the increased risk for cardiovascular pathology in OSA patients have been uncovered by experimental animal models of intermittent hypoxia (IH) mimicking the recurring events of hypoxia-reoxygenation, which have implicated inflammation and oxidative stress as the major culprits (Davis and O’Donnell, 2013; Lavie, 2015; Chopra et al., 2016). However, most of the research focusing on how IH affects the cardiovascular system has revolved around molecular (e.g., oxidative stress, inflammatory cytokines) and cellular (e.g., endothelial dysfunction, inflammatory cells) processes, and only scarce evidence is available on how IH modifies the extracellular matrix (ECM) of cardiovascular tissues (Gileles-Hillel et al., 2014; Castro-Grattoni et al., 2016). Remarkably, how these tissue changes may modify their mechanical properties remains unclear. This is obviously a translationally relevant question, since alterations such as hypertension and right- and left-heart diastolic and systolic dysfunction are modulated by the mechanical properties of the aortic wall and myocardial tissues (Mishra et al., 2013; Xu and Shi, 2014; Egemnazarov et al., 2018). Notwithstanding data showing that oxidative stress may remodel the composition of the ECM of heart tissue (Jacob-Ferreira and Schulz, 2013; Chuang et al., 2014), whether the passive stiffness of the ECM of the left ventricular (LV) myocardium is modified by IH mimicking OSA is unknown. Should this stiffness increase, it could be a mechanism potentially contributing to OSA-associated cardiac dysfunction and failure by limiting either diastolic relaxation and systolic contraction (van Putten et al., 2016; Pandey et al., 2018).

Given that oxidative stress potentially remodels the ECM by modifying its composition and crosslinking (Gilkes et al., 2014; Labrousse-Arias et al., 2017), the aim of this work was to test the hypothesis that hypoxia-reoxygenation events mimicking OSA increase the passive stiffness of the LV myocardium ECM in a murine model. The current study was conducted on decellularized myocardium strips with multiscale approaches being applied to the same tissue samples. Indeed, stiffness at the macroscale was measured using a tensile stretch technique and microscale stiffness was measured by atomic force microscopy (AFM). Furthermore, since aging has been shown to potentially remodel the myocardial ECM (Horn and Trafford, 2016; Meschiari et al., 2017) and thus its stiffness, we conducted studies in both young and aged animals to explore whether the potential effects of IH on myocardial ECM mechanics are modulated by chronological age.

## Materials and Methods

### Animals and Exposures of Intermittent Hypoxia

The study was carried out in 20 young (2-month old) and 20 aged (18-month old) C57BL/6J mice housed in standard cages at the vivarial facilities of the University of Barcelona, with water and food being provided *ad libitum*, while animals were kept in a temperature- and light-controlled room (25°C, 12L:12D). The animal research protocol was approved by the institutional Ethics Committee of Animal Experimentation.

Mice were randomly distributed into 2 groups for IH (10 young and 10 aged mice) and 2 groups for normoxic controls being exposed to room air (RA) (10 young and 10 aged mice). Each group of animals was placed in an experimental setting specially designed for IH exposures mimicking OSA (Almendros et al., 2012). The system was based on a transparent methacrylate box (26 cm long, 18 cm wide, 6 cm high) flushed with air cyclically changing its oxygen content (40 s of normoxic air at 21% O_2_ and 20 s of hypoxic air at 6% O_2_) mimicking a rate of 60 apneas/h, typical of severe OSA. The mice subjected to RA were placed in similar boxes continuously flushed with normoxic air at 21% O_2_. Both exposures were applied for 6 h/day during the light period (10:00–16:00 h) for 6 weeks, with food and water being unrestricted and freely available at all times. At the end of the 6-week exposures, the animals were anesthetized and immediately sacrificed by exsanguination through the abdominal aorta, the hearts were excised, frozen and kept at -80°C for subsequent analysis.

### Measurement of Macroscale Stiffness by Tensile Stretching

The hearts were thawed at room temperature, the left ventricle wall was excised and a strip of ∼7 mm × 1 mm × 1 mm was cut with a scalpel along the long-axis direction. The rest of the myocardial sample was then frozen (-80°C). Each strip was gently dried with tissue paper and its mass (M) measured.

One end of the strip was glued with cyanoacrylate to a small hook attached to the lever of a servo-controlled displacement actuator with an integrated force sensor (300C-LR, Aurora Scientific, Aurora, ON, Canada), which permitted stretching the strip and measuring both the stretched length (*L*) and the applied force (*F*) simultaneously. The other end of the strip was glued to a fixed hook. Measurements were performed inside a bath with PBS at 37°C. The unstretched length (*L*_o_) of the strip was defined as its length at *F* = 0.1 mN and the cross-sectional area (*A*) was computed as

$$A=\frac{M}{\rho \cdot L_{0}}$$

where ρ is tissue density (assumed to be 1 g/cm^3^). The stress (σ) applied to the strip was computed as

$$\sigma =\frac{F}{A}$$

Tissue strain (ε) was defined as

$$\varepsilon =\frac{L}{L_{0}}-1$$

where *L*/*L*_o_ is the stretch. The myocardial strips were initially pre-conditioned by applying 10 stretch cycles at a frequency of 0.2 Hz and maximum stretch of ∼25%, and 10 additional cycles were recorded for analysis. Mechanical properties of the strip at the macroscale were characterized as the average of the last nine stress–strain (σ–ε) curves recorded.

The stiffness of the strip was characterized by the macroscale Young’s modulus (*E*_M_) defined as dσ/dε for a given ε. Stress–strain curves were analyzed with Fung’s model (Fung, 1967) which assumes that *E*_M_ increases linearly with stress as

$$E_{M}=\alpha \cdot \left(\sigma +\beta \right)$$

being α and β the parameters of the model. Then, the stress increases exponentially with strain

$$\sigma =\left(\sigma_{r}+\beta \right)e^{\alpha \left(\varepsilon -\epsilon_{r}\right)}-\beta$$

where σ_r_ and ε_r_ define an arbitrary point of the σ-ε curve. The parameters of Eq. 5 were computed by non-linear least-squares fitting using custom built code (MATLAB, The MathWorks, Natick, MA, United States). The macroscopic stiffness of the strip was characterized as σ and *E*_M_ computed at 20% strain (ε = 0.2).

After native *E*_M_ was assessed, each strip was decellularized to measure *E*_M_ of the ECM. As described elsewhere in detail (Perea-Gil et al., 2015, 2018), myocardial strips were immersed in a 1% sodium dodecyl sulfate (Sigma-Aldrich, Darmstadt, Germany) solution for 48 h, followed by 1% Triton X-100 (Sigma-Aldrich, Darmstadt, Germany) for 24 h, with solutions being replaced every 24 h and constant moderate stirring, and the strips were finally washed for 24 h using phosphate buffered saline (PBS) and gently dried with tissue paper and its mass (M) measured. *E*_M_ of the myocardial ECM was measured as previously described for the native strip.

### Measurement of Microscale Stiffness by Atomic Force Microscopy

After tensile testing, the decellularized myocardial strips were detached from the hooks of the stretching device, immersed in optimal cutting temperature compound (OCT, Sigma-Aldrich, Darmstadt, Germany) and frozen at -8°C. Thin tissue slices (∼12 μm) were obtained by cryosectioning (HM 560, Thermo Fisher Scientific, Waltham, MA, United States) and placed on top of positively charged glass slides. OCT was removed by thawing and washing the samples in PBS at room temperature. Micromechanical properties of the sample were measured in PBS at 37°C pH 7.4 using a custom-built AFM mounted on an inverted optical microscope (TE2000; Nikon, Tokyo, Japan). Measurements were performed with V-shaped silicon nitride cantilevers (0.1 N/m nominal spring constant) ended with a 2.25 μm radius spherical polystyrene bead (Novascan Technologies, Ames, IA, United States). The actual spring constant of the cantilever (*k*) was calibrated by the conventional thermal tune method. The piezo actuator-controlled vertical position of the cantilever (*z*) was measured with strain gauge sensors (Physik Instrumente, Karlsruhe, Germany) and a four-quadrant photodiode (S4349, Hamamatsu, Japan) was employed to measure cantilever deflection (*d*). The relationship between cantilever deflection and photodiode signal was determined from a deflection-displacement (*d*–*z*) curve obtained in a bare region of the glass slide. The force (*F*) applied by the cantilever was computed as

$$F=k\left(d-d_{o}\right)$$

and the indentation (δ) of the sample was computed as

$$\delta =(z-z_{o})-(d-d_{o})$$

being *d*_o_ and *z*_o_ the offset of the deflection and the displacement of the cantilever, respectively, when the tip contacts the surface of the sample. Force indentation curves were analyzed with the Hertz contact model for a rigid sphere indenting an elastic half space

$$F=\frac{4}{3}\frac{E_{m}\cdot \sqrt{R}}{\left(1-\nu^{2}\right)}\delta^{3/2}$$

where *R* is the radius of the tip, *υ* the Poisson’s ratio (assumed to be 0.5) and *E*_m_ the microscale Young’s modulus of the sample. *E*_m_ was computed by fitting Eq. 8 to force-indentation curves by least-squares fitting for a maximum indentation of 0.5 μm using custom built code (MATLAB, The MathWorks, Natick, MA, United States). The micromechanics of each LV myocardium ECM sample was probed in 4 randomly selected zones of the sample. Five force curves (0.5 Hz and 10 μm amplitude) in 4 points randomly selected and separated ∼50–100 μm form each other were recorded in each zone. Micromechanical stiffness of each sample was characterized as the average *E*_m_ computed from the different curves recorded in the sample.

### Collagen Assessment

The extent of fibrosis was assessed from the frozen samples of native myocardial tissue after thawing at room temperature. The collagen content from the mouse cardiac tissue of each group was quantified by a conventional colorimetric assay of the hydroxyproline content (MAK008, Sigma-Aldrich, Darmstadt, Germany). Briefly, dry tissue was weighted and hydrolyzed in 6N HCl 120°C for 3 h and the samples were processed according to the manufacturer instructions. The absorbance was measured at 560 nm in duplicate with a microplate spectrophotometer.

### Statistical Analysis

Data are expressed as mean ± SE. Two-way ANOVA with *post hoc* pairwise multiple comparison with the Student–Newman–Keuls method were performed to compare changes in mechanical parameters and collagen content owing to age and treatment. Statistical significance was considered at *p* < 0.05. The number of viable ECM samples probed in each experiment is indicated in the “Results” section.

## Results

### Tensile Stretch Measurements

**FIGURE 1:**
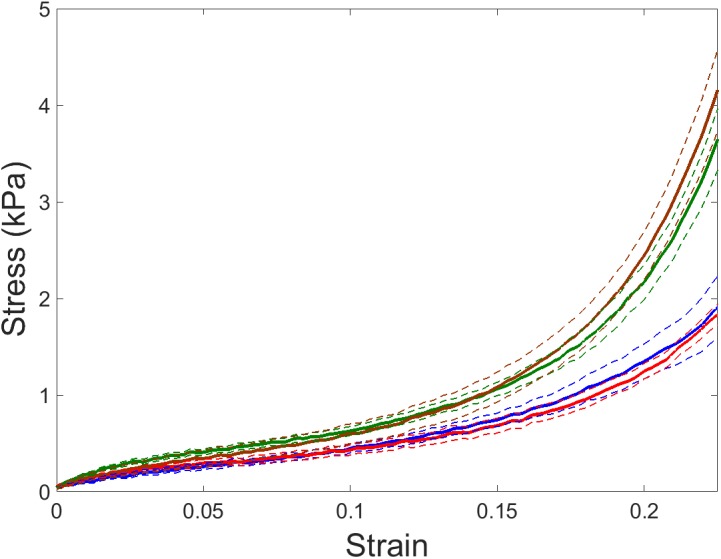
Stress–strain relationships of left ventricular (LV) myocardium ECM. Stress–strain recorded by tensile stretching in ECM myocardium strips of young and aged mice subjected to room air (blue and red lines, respectively) and intermittent hypoxia (IH) exposures mimicking obstructive sleep apnea (OSA) (green and brown lines, respectively). Data are mean (solid lines) ± SE (dashed lines).

**FIGURE 2:**
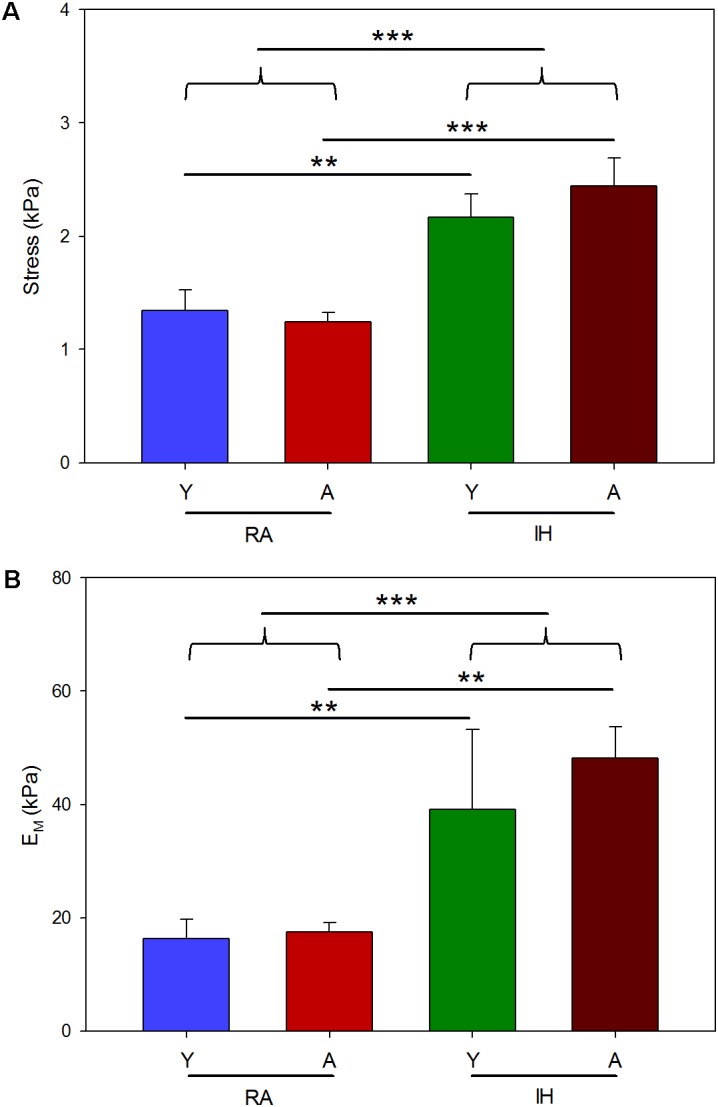
Macroscale stiffness of LV myocardium ECM computed from the stress–strain relationship curves. Stress **(A)** and macroscale Young’s modulus (*E*_M_) **(B)** computed at 20% strain from young (Y) and aged (A) mice subjected to room air (RA) and IH mimicking OSA. Data are mean ± SE. ^∗^, ^∗∗^, ^∗∗∗^ denote *p* values less than 0.05, 0.01 and 0.001, respectively.

Left ventricular myocardium ECM strips exhibited a marked strain hardening behavior showing an approximately exponential stress–strain relationship (**Figure 1**). At 20% strain, ECM strips from young mice breathing room air (*n* = 7) showed σ = 1.35 ± 0.18 kPa and *E*_M_ = 16.29 ± 3.45 kPa. Similar results were found in aged mice (σ = 1.24 ± 0.08 kPa and *E*_M_ = 17.49 ± 1.59 kPa; *n* = 7). IH exposures induced a ∼1.5-fold increase in σ (**Figure 2A**) and ∼2.5-fold increase in *E*_M_ (**Figure 2B**) both in young (σ = 2.17 ± 0.21 kPa and *E*_M_ = 39.17 ± 14.10 kPa; *n* = 5) and aged mice (σ = 2.44 ± 0.25 kPa and *E*_M_ = 48.13 ± 5.63 kPa). Two-way ANOVA analysis revealed a significant effect of IH both in stress (*p* < 0.001) and in macroscale Young’s modulus (*p* < 0.001). In contrast, no significant effect of age emerged on σ (*p* = 0.757) or *E*_M_ (*p* = 0.164).

As expected from previously reported data, stiffness measured in native and decellularized LV myocardial strips were very close and well correlated: taking into account the whole samples *E*_M_ was 30.46 ± 5.38 kPa and 29.68 ± 4.23, respectively (*p* = 0.784 in paired *t*-test), coefficient of correlation *r* = 0.852 (*p* < 0.001). Similar agreement was found when comparing σ between native (1.77 ± 0.14 kPa) and decellularized (1.70 ± 0.20 kPa) strips (0.606 paired *t*-test; coefficient of correlation *r* = 0.812, *p* < 0.001).

### AFM Measurements

**FIGURE 3:**
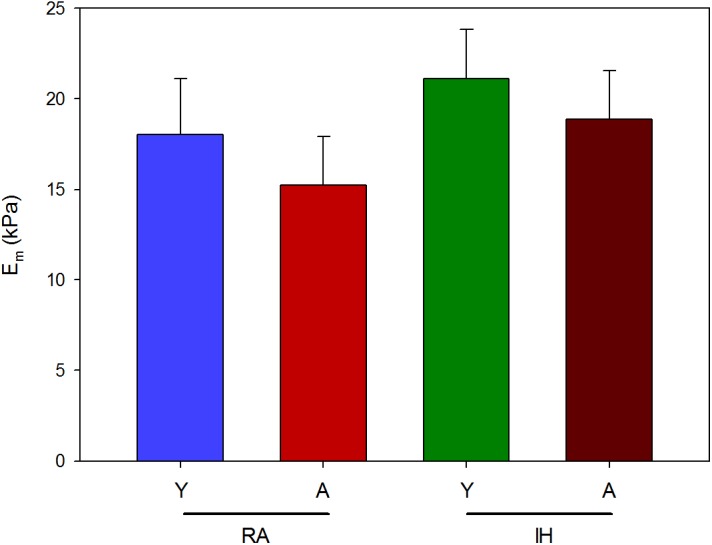
Microscale stiffness of LV myocardium ECM. Microscale Young’s modulus (*E*_m_) computed by AFM from young (Y) and aged (A) mice subjected to room air (RA) and IH mimicking OSA. Data are mean ± SE.

Microscale Young’s modulus computed from AFM measurements was similar to *E*_M_ computed by tensile testing at 20% stretch. Young (*n* = 6) and aged (*n* = 6) mice breathing room air showed *E*_m_ of 18.03 ± 3.07 kPa and 15.25 ± 2.68 kPa, respectively. Slightly higher values of *E*_m_ were observed when young (21.12 ± 2.70 kPa; *n* = 6) and aged (18.89 ± 2.65 kPa; *n* = 6) mice were subjected to IH (**Figure 3**). No statistically significant effects of age (*p* = 0.378) and IH (*p* = 0.240) on ECM stiffness were found when the latter was measured at the microscale level.

### Collagen Content

**FIGURE 4:**
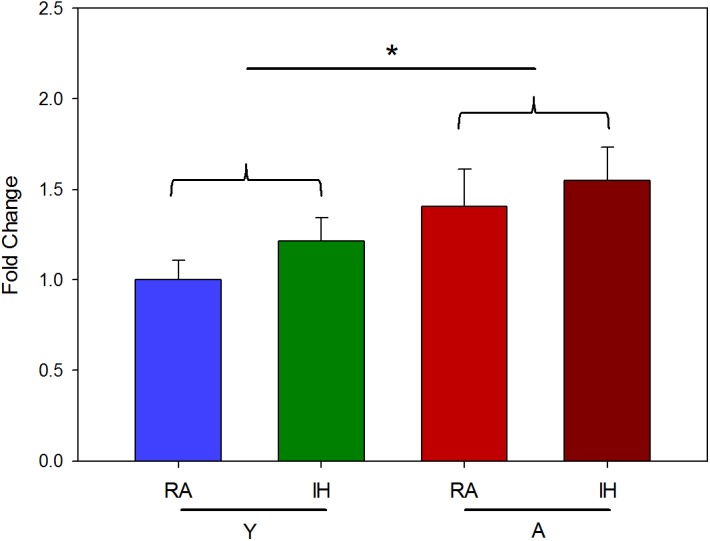
Collagen content of LV myocardium ECM. Hydroxyproline content (fold-change respect the Y-RA group) measured in young (Y) and aged (A) mice subjected to room air (RA) or IH mimicking OSA. Data are mean ± SE. ^∗^*p* < 0.05.

Collagen content (expressed in fold-change respect to the mean of RA-young group) was 1.00 ± 0.11 (RA, *n* = 7) and 1.21 ± 0.13 (IH, *n* = 8) for young mice and 1.41 ± 0.20 (RA, *n* = 8) and 1.55 ± 0.19 (IH, *n* = 10) for aged mice (**Figure 4**). Two-way ANOVA analysis found significant effects of age (*p* < 0.05), but no significant effect of IH exposures (*p* = 0.203).

## Discussion

This multiscale mechanical study reveals for the first time that IH mimicking OSA differentially affects the macro- and micromechanical properties of the LV myocardium ECM. Indeed, mice subjected to IH exhibited marked increases in ECM stiffness measured at the macroscale by tensile stretching. In contrast, no significant changes were apparent in microscale ECM stiffness measured by AFM. Interestingly from a translational perspective, the observed effect of IH on ECM stiffness was similar among young and aged mice.

The current study was carried out using a widely accepted animal model of OSA that realistically mimics the episodic events of hypoxia-reoxygenation that characterize this highly prevalent medical condition. Indeed, the high frequency (60 events/h) and magnitude [arterial oxygen desaturation with nadir values of 60–70% (Torres et al., 2015)] of the IH events imposed to the mice, as well as the duration of daily exposures, were sufficient to warrant the designation of severe, but in no way unrealistic since they remarkably overlap and recapitulate the oxygenation patterns seen in patients with severe OSA (Ruehland et al., 2009; Lloberes et al., 2011). Such an IH paradigm has been extensively employed to reproduce the different morbid consequences of OSA: cardiovascular (Ramos et al., 2014), neurocognitive (Gozal et al., 2017), metabolic (Moreno-Indias et al., 2015), reproductive (Torres et al., 2014), and malignancies (Campillo et al., 2017). Interestingly, and contrasting with the majority of experimental research models that have traditionally used only young animals to mimic diseases occurring over a wide range of chronological ages, we here included both young and aged mice whose ages would be equivalent to ∼20 year old and 60–65 year old humans, respectively (Flurkey et al., 2007). This is particularly important, considering that older age may be an important modifier of the phenotypic expression of OSA-associated morbidity (Lavie and Lavie, 2009).

The ECM is a major determinant of the passive macromechanical properties of the LV myocardium as previously reported (Perea-Gil et al., 2018) and when observing the very close values of *E*_M_ and σ we found when comparing native and decellularized samples. Consequently, macroscale ECM stiffening could result in diastolic dysfunction characterized by increased LV end-diastolic pressures (Zile et al., 2004; Rommel et al., 2016). On the other hand, micromechanical properties of the ECM are a key factor of the cell-matrix crosstalk which mediates critical cellular processes such as contractility, proliferation and differentiation (Alcaraz et al., 2003; Ng et al., 2012; Yeh et al., 2012; Macrí-Pellizzeri et al., 2015). Consequently, LV myocardial ECM was studied using a multiscale approach to gain a thorough insight into changes in its mechanical properties as a consequence of *in vivo* IH exposures. It has been recently reported in a porcine model of heart regeneration that structure, micro- and micromechanical properties of LV myocardium tissues are preserved after decellularization (Perea-Gil et al., 2015, 2018). Therefore, all measurements were performed in decellularized tissues to facilitate sample preparation for AFM measurements, and to compare micro- and micromechanics under the same conditions and in the same tissue samples such as to minimize inter-sample variability.

Macroscale mechanics was studied in LV myocardium ECM strips by uniaxial tensile testing. This technique enables direct characterization of sample stiffness, thereby avoiding the geometric assumptions required when tissue mechanics are determined from pressure-volume measurements in the whole ventricular cavity (Voorhees and Han, 2015). Strip uniaxial testing also avoided some difficulties presented by echocardiography which is the cornerstone tool to assess cardiac morphology and function in human. Indeed, values of heart size and beating rate in mice pose technical limitations that makes difficult a complete functional and anatomical study in these animals (Fayssoil and Tournoux, 2013; Schnelle et al., 2018). Owing to the small size of the mouse heart, tensile testing was performed in strips cut along the LV longitudinal axis. However, it is expected that stiffness changes observed by applying longitudinal stretch testing are representative of changes in bulk tissue stiffness since no significant differences between longitudinal and circumferential loading directions of macroscale stiffness of LV myocardium ECM have been reported (Quinn et al., 2016). Given the marked strain-hardening behavior of the myocardium (**Figure 1**), macroscopic stiffness was characterized as the values of σ and *E*_M_ when these were computed at 20% strain which is in the strain range experienced by the LV wall under physiological heart functioning conditions (Park et al., 2016). Macroscopic ECM stiffness exhibited by mice breathing air o among those subjected to IH are comparable with the values recently reported in swine (Perea-Gil et al., 2018). On the other hand, AFM is a well-suited technique for micromechanical measurements of tissue slices at a length scale of ∼1 μm, which is the length scale at which cells sense the mechanical properties of their mechanical niche (Andreu et al., 2014). The values of microscale stiffness found in this study are also in the range of those previously reported in mice and swine (Andreu et al., 2014; Perea-Gil et al., 2018). ECM stiffness at the macroscopic scale is determined by the local micromechanics of the matrix and its structural 3D assembly. The lack of significant changes in stiffness at the microscale (**Figure 3**) suggests that the IH-induced stiffening of the LV myocardium ECM at the macroscale (**Figure 2**) is principally determined by 3D structural remodeling of the matrix (e.g., different fibers density, orientation and cross-linking). However, better understanding of the detailed relationships between micro- and macroscale stiffness in non-homogeneous, anisotropic biological soft tissues – which are measured by very different experimental approaches and theoretical assumptions – requires further future research (McKee et al., 2011).

The results indicating that aging does not play a significant role in modifying the passive stiffness of the LV myocardium ECM both in normoxic conditions and under IH (**Figures 2**, **3**) have translational relevance for OSA. The age span of the mice in this study encompassed the range of young to late middle age humans. We excluded senescent ages (>70 years in humans) since the phenotype and mortality rates differ in advanced aging (Lavie et al., 2005) and it is unclear how OSA naturally progresses in senescent ages (Sforza et al., 2012). The lack of aging effect found in myocardial ECM stiffness is consistent with the values for end-systolic and end-diastolic LV elastances reported in humans, which showed only very modest increases over the 50–90 year old age range (Redfield et al., 2005; Borlaug et al., 2013). The amount of collagen found in fresh myocardium tissues, usually considered a global index of fibrosis, was slightly higher (by 41%) in the older normoxic mice, a finding that is consistent with data previously reported for aged mice (Derumeaux et al., 2008; Chiao et al., 2012). It is of note that collagen abundance results from a balance between increased collagen deposition and cross-linking, and increased ECM degradation due to increased expression of matrix metalloproteinases with aging (Meschiari et al., 2017). However, the relationship between ECM stiffness and collagen concentration is not direct (Quinn et al., 2016), since factors such as collagen distribution, different collagen types, and collagen cross-linking should be taken into consideration, while also accounting for the contribution of other important ECM components in determining ECM stiffness (Collier et al., 2012; Lopez et al., 2012). In contrast with the lack of effects of aging on myocardial ECM mechanics, IH markedly increased ECM macroscopic stiffness. This finding should be attributed to changes induced by hypoxia-reoxygenation on the different components and structure of the ECM. Although the data available in this regard are scarce, it has been recently reported that hypoxia reduced heart fibrosis after myocardial infarction in mice (Nakada et al., 2017), and that upregulation of lysyl oxidase, an enzyme contributing to changes in the structure of collagen and elastin fibers, is associated with enhanced oxidative stress that promotes structural alterations and vascular stiffness, and could play a potential role in cardiac remodeling (El Hajj et al., 2017; Galán et al., 2017; Varona et al., 2017). Interestingly, increased expression of lysyl oxidase has been reported in OSA patients (Mesarwi et al., 2015).

The finding that the macroscopic stiffness of the LV myocardium ECM is increased by IH mimicking OSA may provide insights into a relevant cardiovascular consequence of this sleep breathing disorder, namely heart failure (HF) (Baguet et al., 2012). Indeed, OSA is clinically associated with HF (Schulz et al., 2007), with OSA increasing the risk of HF by 2.2-fold (Shahar et al., 2001). Moreover, experimental data have shown that application of chronic IH mimicking OSA results in adverse LV remodeling (Hayashi et al., 2011) and in cardiac dysfunction typical of HF, such as increases in end-diastolic volume, and decreases in ejection fraction (Wei et al., 2016). However, ECM myocardial mechanics is only one among several potential contributing factors by which OSA can induce HF (e.g., increased sympathetic activity, endothelial dysfunction, systemic inflammation, oxidative stress, metabolic anomalies, and immune alterations) (Baguet et al., 2012; Farré et al., 2018). In fact, the complexity and sometimes counteracting effects of the pathways activated and propagated by IH may account for some of the conflicting experimental findings concerning cardiac dysfunction in OSA (Naghshin et al., 2009). Notwithstanding, there is clear evidence that, together with myocytes, the ECM plays a relevant role in determining the passive mechanical properties of the myocardium (Borlaug, 2014), and hence in modulating diastolic dysfunction (Zile et al., 2004; Rommel et al., 2016). Accordingly, an increase in ECM passive bulk stiffness, such as observed as a consequence of IH, would negatively contribute to end-diastolic volume. On the other hand, studies carried out in cardiomyocytes seeded in polyacrylamide gels reported that increased substrate stiffness disturbs normal cardiomyocyte differentiation and maturation from progenitor cells (Engler et al., 2008), and decreases their contractile activity (Dasbiswas et al., 2015), suggesting that IH would promote systolic dysfunction through increase in ECM elastance. Consequently, the stiffening of the ventricular myocardial ECM observed in this study when the animals were chronically subjected to IH reinforces the assumption that one of the factors contributing to HF in OSA would be cardiac matrix remodeling.

This work was focused on the effects of IH mimicking sleep apnea on the biomechanics (passive stiffness) of LV myocardial ECM. To this end, we used macro- and microscale techniques (tensile and AFM, respectively) to answer the main question. Given our current limited knowledge on myocardial ECM remodeling by the hypoxia-reoxygenation events experienced by patients with OSA, the present biomechanics study at the ECM level opens the opportunity for future studies (from the biochemical mechanisms at molecular scale to heart function at functional scale *in vivo*) to explore the potential mechanisms involved and the functional consequences in detail.

## Conclusion

In conclusion, exposures to chronic IH mimicking severe OSA exerted a differential effect on the micro- and macromechanics of the LV myocardium ECM: whereas the local Young’s modulus measured by AFM remained unaltered, the bulk elastance increased considerably, suggesting 3D remodeling of the mesh network in the matrix scaffold. Increases in the macroscopic stiffness by IH, which were age-independent, would be anticipated to contribute to the cardiac dysfunction frequently observed among OSA patients. The novel results of this study add further support to the notion that multiscale studying matrix mechanics may contribute to interpreting basic mechanisms in cardiorespiratory diseases.

## Ethics Statement

The experimental procedures were approved by the Ethics Committee of Animal Experimentation of the University of Barcelona following the local and European regulations in force.

## Author Contributions

NF and DN conceived the work and designed the experiments. JO carried out most of the experiments and data analysis. BF, IJ, and MT contributed to the experimental work. RF, DN, DG, IA, IJ, JO, and BF analyzed and interpreted the data. All authors discussed the work and contributed to writing the final version of the manuscript drafted by NF and DN.

## Conflict of Interest Statement

The authors declare that the research was conducted in the absence of any commercial or financial relationships that could be construed as a potential conflict of interest.
